# Clinical features, laboratory characteristics, and outcome of ETP and *TCRA/D* aberrations in pediatric patients with T-acute lymphoblastic leukemia

**DOI:** 10.1186/s43046-023-00176-1

**Published:** 2023-06-12

**Authors:** Mona S. El Ashry, Enas Radwan, Mona S. Abdellateif, Omar Arafah, Naglaa M. Hassan

**Affiliations:** 1 Clinical Pathology Department, National Cancer Institute, Cairo University, Cairo, Egypt; 2 Medical Biochemistry and Molecular Biology, Cancer Biology Department, National Cancer Institute, Cairo University, Cairo, Egypt; 3 Pediatric Oncology Department, National Cancer Institute, Cairo University, Cairo, Egypt

**Keywords:** Early T-cell precursor, Child, T-ALL, T-cell receptor

## Abstract

**Background:**

T-cell acute lymphoblastic leukemia (T-ALL) is an aggressive malignancy with few accepted prognostic factors that limit the efficiency of therapy. The aim of the current study was to assess the clinical and laboratory features of T-cell receptor (TCR) aberrations and early T-cell precursor (ETP) subtype as well as their outcome to therapy.

**Methods:**

Sixty-three newly diagnosed pediatric T-ALL patients were assessed for the ETP status using immunophenotyping. Screening of TCRA/D aberrations was done by fluorescent in situ hybridization (FISH). The data were correlated to the patients’ clinical features, response to treatment, and survival rates.

**Results:**

Seven patients (11%) had ETP-ALL. The ETP-ALL patients were older (*P* = 0.013), presented with lower white blood cell (WBC) count (*P* = 0.001) and lower percentage of peripheral blood (PB) blast cells (*P* = 0.037), more likely to have hyperdiploid karyotype (*P* = 0.009), and had been associated with TCRA/D gene amplification (*P* = 0.014) compared to other T-ALL patients. Of note, the same associations had been significantly observed in patients with TCRA/D gene amplification. Patients with TCRA/D amplification frequently coincided with TCRβ aberrations (*P* = 0.025). TCR-β aberrations were significantly associated with negative MRD at the end of induction compared to TCR-β-negative patients. There was a nonsignificant trend of ETP-positive cases to have lower overall survival (OS) (*P* = 0.06). Patients with TCR aberrations had no significant differences regarding disease-free survival (DFS) or OS rates compared to those with normal TCR.

**Conclusion:**

ETP-ALL patients tend to have increased mortalities. There was no significant impact of TCR aberrations on the survival rates of the patients.

**Supplementary Information:**

The online version contains supplementary material available at 10.1186/s43046-023-00176-1.

## Introduction

T-cell acute lymphoblastic leukemia (T-ALL) is an aggressive malignancy of the thymocytes caused by the accumulation of genomic lesions that affect the development of T cells. It mainly affects children, commonly boys, accounting for 15% of pediatric ALL and 20% of adult ALL [1–3]. Historically, T-ALL has been associated with poor outcome; however, with more contemporary therapy, outcomes for T-ALL and B-cell ALL become similar [1, 4].

Although there are validated prognostic factors in T-ALL, e.g., response to treatment including induction remission status and minimal residual disease (MRD) levels, there is controversy regarding the prognostic significance of the other factors including early T-cell precursor (ETP) phenotype and recurrent genetic aberrations [1, 4].

The ETP subtype, occurring in 15% of childhood T-ALL cases, was first identified within pediatric T-ALL cases based on its unique immunophenotypic and genetic features [1, 4, 5]. The hypothesis regarding the cell of origin and its leukemogenic pathways is still debated. Although having characteristics of T-cell lineage commitment, these cells continue to have the potential for myeloid/dendritic cell differentiation. Therefore, it is considered as a stem cell leukemia at the crossroads of the lymphoid and myeloid fates [4, 6].

As such, ETP-ALL immunophenotype was defined as follows: (1) absent (< 5% positive cells) CD1a and CD8 expression; (2) absent or dim (< 75% positive cells) CD5 expression; and (3) expression (> 2 5% positive cells) of 1 or more myeloid (CD11b, CD13, CD33, CD117) or stem cell (CD34, HLA-DR) markers [1, 5, 6]. Initially, ETP status was associated with high rates of induction failure, early relapse, and poor overall survival. However, in more recent reports, ETP status has lacked prognostic significance; therefore, the prognostic impact of ETP remains a subject of debate [1, 4].

While several genetic aberrations associated with T-ALL pathogenesis had been identified [5, 6], few of them have been reproducibly associated with prognosis, and none is prospectively used in risk stratification [4]. There are four major mechanisms known to cause aberrant expression of transcription factors in T-ALL including the following: (1) chromosomal translocations involving one of the TCR genes, (2) chromosomal rearrangements with other regulatory sequences, (3) duplication/amplification of the transcription factor, and (4) mutations or small insertions generating novel regulatory sequences acting as enhancers [3].

Translocations and inversions involving the TCR genes are now recognized as the oncogenic hallmark of T-ALL. TCR translocations occur in 35% of cases, as ascertained by FISH and PCR analyses, with almost 50% going undetected by chromosome banding analysis. The TCR aberrations include the TCR alpha/delta chain (*TCRA/D*) which is located at 14q11.2 (in 17% of T-ALL cases), the TCR beta chain (*TCRβ*) which is located at 7q34, and the TCR gamma chain (*TCRδ*) which is located at 7p14 [7].

Upon characterizing TCR rearrangements, about 30 partner oncogenes have been identified and proved to be important for T-ALL oncogenesis [7–9]. The overexpression of TAL1 (chromosome 1p32) and LMO1/LMO2 (11p13) expression are caused by rearrangements to the *TCRA/D* chain in 3–9% of pediatric T-ALL patients, while overexpression of HOX11 (TLX1) (10q24) is caused by rearrangements to the promoters of the *TCRβ* or *TCRA/D* chains in 30% of adult T-ALL [7, 10].

Current treatment of pediatric T-ALL consists of high-intensity combination chemotherapy which results in a very high overall survival (OS) [11]. Unfortunately, this treatment comes with significant side effects that should not be underestimated [12, 13]. Moreover, the occurrence of relapse is a challenging matter, as it is observed in up to 20% of cases and is often refractory to chemotherapeutics [14]. In addition, the development of targeted therapy for T-ALL might be controversial, given that it was demonstrated that TCR-positive T-ALL cells can be targeted by mimicking thymic negative selection [13].

Accordingly, in this study, we aimed at investigating the characterization of ETP-ALL and *TCRA/D*-positive cases in pediatric T-ALL with special emphasis on the clinical features associated with those cases and their impact on the outcome of the patients.

## Patients and methods

### Patients

The present retrospective study included 63 newly diagnosed pediatric T-ALL patients who were diagnosed during the period from January 2016 to July 2020. All patients were presented to the Outpatient Clinic of the Paediatric Oncology Department, National Cancer Institute (NCI), Cairo University, Egypt.

## Methods

The diagnosis of T-ALL was based on the morphological examination of the peripheral blood (PB) and bone marrow (BM) smears, cytochemistry, immunophenotyping by flow cytometry, conventional cytogenetics, and cerebrospinal fluid (CSF) analysis. In addition to the initial workup such as chest radiographs (X-ray or CT chest) and abdominal ultrasonography.

### Flow cytometry analysis

Immunophenotyping was performed on either BM or PB samples using a wide panel of monoclonal antibodies (Abs) for initial lineage assignment, according to the standard techniques. Fluorescein-labelled mouse monoclonal antibodies (MPO, CD13, CD33, CD117, CD64, CD14, and CD11c) were used to identify myeloid lineage. B-lymphoid cells were identified by using CD19, CD10, CD20 cytoplasmic μ chain, kappa, Lambda, and cytCD22/cytCD79a), while T-lymphoid antigens were identified using CD3, CD2, CD5, CD7, CD4, CD8, TDT, and CD1a in addition to C45, CD34, and HLA-DR. The acquisition was done on Navios 6 colors flow cytometry (Beckman Coulter, Miami, FL, USA).

The diagnosis of T-phenotype was considered only upon the expression of the lineage-specific marker (surface/cytoplasmic CD3). Patients were classified into immunophenotypic subgroups: early T-cell precursor phenotype (ETP) (CD1a^−^, CD8^−^, CD5^dim^/^−^, positivity for ≥ 1 myeloid/stem cell-related markers, i.e., CD34, CD117, CD13, CD33 or HLADR), early T-phenotype (CytCD3^+^, CD1a^−^, SCD3^−^, CD4^−^, and CD8^−^), intermediate T-phenotype (CytCD3^+^, CD1a^+^, and/or co-expression of CD4 and CD8), and late T-phenotype (CytCD3^+^, SCD3^+^, CD4^+^, or CD8^+^).

### Cytogenetic analysis

The pre-treatment diagnostic BM samples were subjected to conventional karyotyping using G-banded metaphase cells from unstimulated 24- and 48-h cultures following the standard techniques. In most of the cases, at least 20 metaphases were analyzed using an IKAROS imaging system (MetaSystems, Altlussheim, Germany). The karyotypes were interpreted using the International System for Human Cytogenetic Nomenclature (ISCN 2016 [15] and 2020 [16]).

For the detection of *TCRA/D* rearrangements, amplifications, and deletions, fluorescence in situ hybridization (FISH) was performed using XL *TCRA/D* (MetaSystems, Altlussheim, Germany) according to the manufacturer’s instructions. A minimum of 200 interphase nuclei and 10 metaphases were analyzed using a fluorescence microscope (AxioImager.Z1 mot, Carl Zeiss Ltd., Hertfordshir, UK) equipped with appropriate filter sets. Image capture and processing were performed using an ISIS imaging system (MetaSystems, Altlussheim, Germany).

### Management of the patients

All children received total XV protocol (modified from St. Jude total XV protocol) [17]. Patients with T-ALL were classified into either standard or high-risk based on response to treatment assessed morphologically and by minimal residual disease levels measured by flowcytometry on day 15 of start of chemotherapy and at the end of induction therapy (day 42).

The treatment protocol consisted of three phases, induction of remission, consolidation, and maintenance [18]. The induction phase (6 weeks) included prednisone, vincristine, doxorubicin, L-asparaginase, cyclophosphamide, cytarabine, and mercaptopurines. Age-dependent triple intrathecal chemotherapy was given on days 1, 4, 8, 12, 15, and 22 regardless of the initial CNS status in all T-cell patients. Regarding the patients who did not achieve morphological CR at day 15 (≥ 5% BM blasts) or had minimal residual disease (MRD) ≥ 1% by flow cytometry, they received additional 3 doses of L-asparaginase, while the patients who achieved morphological CR at day 42 induction (< 5% BM blasts) or had *MRD* < 1% were considered a standard risk and received the consolidation phase. Patients who did not achieve morphological CR at day 42 induction (≥ 5% BM blasts) or had *MRD* ≥ 1% were considered high risk and received re-intensification therapy before the consolidation phase and were considered for allogeneic hematopoietic stem cell therapy (HSCT) if they have HLA-matched sibling donor. The consolidation therapy (8 weeks) consisted of 4 cycles of high-dose MTX (HDMTX) and 56 days of 6MP followed by maintenance treatment duration for 120 weeks for girls and 146 weeks for boys (supp. 1).

### Statistical analysis

Data management and analysis were performed using SPSS, version 22 (IBM, Armonk, NY, USA). Qualitative data were presented as numbers and percentages, while the quantitative data were presented as median and interquartile ranges (IQR) according to the appropriate normality test. The comparison between groups was performed using chi-square test and/or Fisher exact test which is appropriate. The Mann–Whitney test was used for comparing numerical variables between two groups. Survival analysis was done using the Kaplan–Meier test, and comparison between survival curves was done using the log-rank test. Overall survival (OS) was calculated from the date of diagnosis until the date of last follow-up or death due to any cause. Disease-free survival (DFS) was calculated from the date of CR until the date of relapse, death during complete remission, or second malignant neoplasms. All tests of hypotheses had been conducted at the alpha level of 0.05, with a 95% confidence interval.

## Results

### Patient’s characteristics

The current study included 63 pediatric patients with de novo T-ALL with a median age of 7 (range of 1 to 18) years old. Males represented 69.8% (44/63), and females were 30.2% (19/63). Seventeen patients (26.9%) had intermediate T-phenotype, while 26 (41.3%) had late T-phenotype. The median of modal chromosome number (MCN) was 46 (range: 41–91); 11/63 patients (17.5%) had hyperdiploidy, while 6/63 (9.5%) had hypodipiody.

Abnormal cytogenetics were detected in 68.3% (43/63) T-ALL patients. Structural changes were more common than numerical ones with 62.8% (27/43) and 20.9% (9/43) of abnormal cases, respectively, while 16.3% (7/43) of abnormal cases displayed both structural and numerical anomalies. Baseline demographic, clinical, and laboratory characteristics are shown in Table 1.

**Table 1:** Clinical and laboratory characteristics of the study cohort

Variable		Frequency	Percent	Variable		Frequency	Percent
**Age**: median (range)		7 (1–18)		**TLC**: median (range)		187.6 (2–967)	
**HB**: median (range)		7.7 (4–14.5)		**Platelets**: median (range)		48 (8–693)	
**PB blast**: median (range)		82 (0–99)		**BM blast**: median (range)		89 (0–99)	
**MCN**: median (range)		46 (range: 41–91)		**MCN**	Normal	46	73.0
**Sex**	Male	44	69.8		Hypodiploidy	6	9.5
	Female	19	30.2		Hyperdiploidy	11	17.5
**Initial CNS**	CNSI	48	76.2	**IPT diagnosis**	T intermediate	17	27.0
					T early	16	25.4
	TLP	13	20.6		T late	23	36.5
	CNSIII	2	3.2		ETP	7	11.1
**BM cellularity**	Hypercellular	51	81	**CD1**	Negative	12	19.0
	Normocellular	12	19		Positive	51	81.0
**CD117**	Negative	50	79.4	**CD7**	Negative	26	41.3
	Positive	13	20.6		Positive	37	58.7
**CD34**	Negative	53	84.1	**CD3 surface**	Negative	53	84.1
	Positive	10	15.9		Positive	10	15.9
**CD2**	Negative	33	52.4	**CD5**	Negative	44	69.8
	Positive	30	47.6		Positive	19	30.2
**HLA-DR**	Negative	35	55.6	**TDT**	Negative	8	12.7
	MHCII	28	44.4		Positive	55	87.3
**Complex** (presence of 3 or more cytogenetics abnormalities)	Negative	53	84.1	**Cytogenetics**	Normal	20	31.7
	Positive	10	15.9		Abnormal	43	68.3
**MRD15**^a^	< 0.01	10	21.7	**MRD42**	< 0.01	15	35.7
	≥ 0.01	36	78.3		≥ 0.01	27	64.3
**Induction outcome**	CR	52	82.5	**Relapse**	Negative	55	87.3
	Induction death	11	17.5		Positive	8	12.7
**Early death**	Negative	52	82.5	**Death**	Negative	36	57.1
	Positive	11	17.5		Positive	27	42.9

*BM* bone marrow, *CD* cluster of differentiation, *CNS* central nervous system, *CR* complete remission, *IPT* immunophenotyping, *HLA-DR* human leukocyte antigen — DR isotype, *MCN* modal chromosomal number, *MRD* minimal residual disease, *BP* peripheral blood, *TLC* total leukocyte count, *TDT* terminal deoxynucleotidyl transferase

^a^Missing numbers for MRD were due to the adequacy of the samples and the presence of leukemia-associated immunophenotypes

### Frequency of ETP and TCR aberrations in pediatric T-ALL

**Fig. 1:**
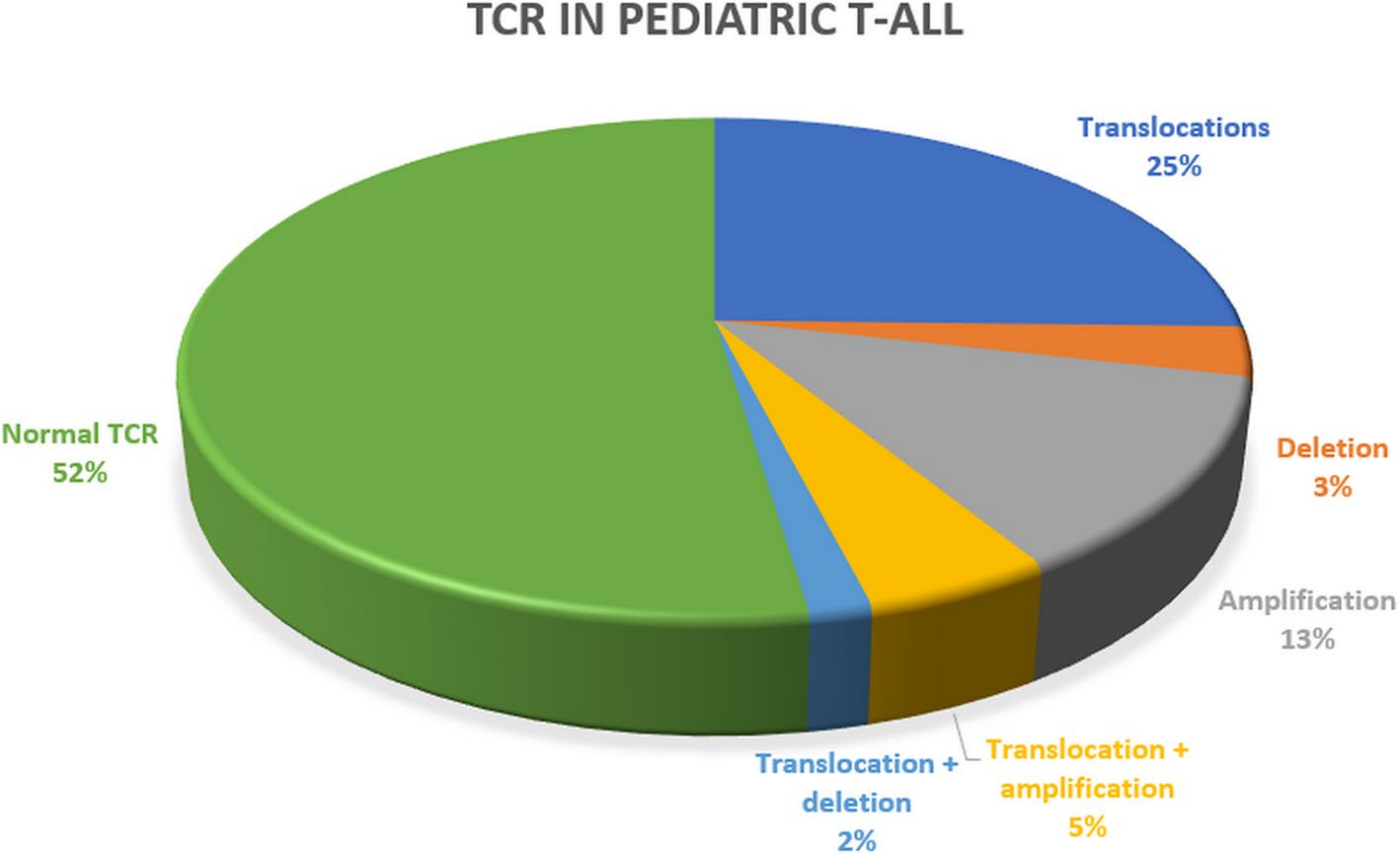
Frequency of TCR/D, TCR-β, and ETP alterations in pediatric T-ALL cases

The ETP was observed in 7/63 (11.1%) of the patients. Upon screening by FISH, 4 types of *TCRA/D* aberrations were found among the 63 pediatric patients: (I) *TCRA/D* translocations in 20/63 (31.7%) patients, (II) *TCRA/D* amplifications in 11/63 (17.5%) patients, (III) *TCRA/D* deletions in 3/63 (4.8%) patients, and (IV) collective *TCRA/D* abnormalities, including the previously mentioned 3 aberrations, in 30/63 (47.6%) patients (Fig. 1).

*TCRA/D* translocation was the sole structural abnormality in 15/20 (71%) patients, while 5/20 (29%) harbored additional structural and/or numerical aberrations. The identified partners were chromosome 8 (*n* = 2.10%), chromosome 11 (*n* = 2.10%), and chromosome 6 and chromosome 12 (one case each), while 14 cases were of unidentified partner chromosome. Of note, 3/20 (15%) patients had concurrent *TCRA/D* amplification, while 1 patient had *TCRA/D* deletion.

Out of 11 cases with *TCRA/D* amplification, 7 cases (63.6%) gained one or more copy of chromosome 14 in hyperdiploid karyotypes, while 4 cases (36.3%) showed *TCRA/D* duplications. Notably, all cases with *TCRA/D* deletions had monosomy 14 in hypodiploidy karyotypes. Aberrations involving *TCR-β* were found in 13/63 patients (20.6%); 9/13 (69.2%) showed *TCR-β* structural abnormalities of which 3 (23%) cases with t(1;7)(p32;q34), while 4/13 (30.8%) gained extra-copy of *TCR-β* in hyperdiploid karyotypes.

### Association of ETP with ALL patients’ characteristics

Based on the immunophenotype, 7/63 (11.1%) patients were classified as ETP and 56/63 (88.9%) as non-ETP. Patients with ETP were significantly older than the other T-ALL patients (*P* = *0.013*). Initial WBC count less than 50,000/µl was seen significantly in 71.4% of ETP compared to 20.3% of non-ETP (*P* = *0.001*). The ETP patients showed significantly lower percentage of PB blast cells compared to non-ETP patients [50% (0–85%) vs 85% (0–99%); respectively, *P* = *0.037*]. Of note, all patients (100%) with ETP-ALL had lymph node enlargement at presentation, but the relation was not statistically significant.

Regarding cytogenetics, patients with ETP-ALL were more likely to have hyperdiploid karyotype compared to non-ETP patients (57.1% vs 12.5%, respectively, *P* = *0.009*) with a nonsignificant trend to have complex karyotypes (42.9% vs 12.5%, respectively, *P* = *0.073*, Table 2).

**Table 2:** Clinical features, laboratory characteristics, and outcome of ALL in relation to ETP subtype

Parameters		ETP (*N* = 7, 11.1%)	Non-ETP (*N* = 56, 88.9%)	*p-value*
**Age**	Median (range)	13 (4–18)	6 (1–18)	**0.013**
**WBC** (× 10^9^/L)	Median (range)	12.9 (3.2–100)	240 (1.7–967)	**0.001**
**Hemoglobin** (g/dl)	Median (range)	8.7 (5.9–14.4)	7.6 (4–14.5)	0.248
**Platelet count** (× 10^9^/L)	Median (range)	64 (8–224)	46 (9–693)	0.306
**PB blast (%)**	Median (range)	50 (0–85)	85 (0–99)	**0.037**
**BM blast (%)**	Median (range)	88 (28–92)	90 (0–99)	0.230
**Gender**	Male	5 (71.4%)	39 (69.6)	0.923
	Female	2 (28.6%)	17 (30.4)	
**CD34**	Negative	5 (71.4)	48 (85.7)	0.306
	Positive	2 (28.6)	8 (14.3)	
**Cytogenetics**	Normal	1 (14.3)	19 (33.9)	0.415
	Abnormal	6 (85.7)	37 (66.1)	
**MCN**	Diploid	2 (28.6)	44 (78.6)	**0.009**
	Hypodiploid	4 (57.1)	7 (12.5)	
	Hyperdiploid	1 (14.3)	5 (8.9)	
**Complex karyotypes**	Negative	4 (57.1)	49 (87.5)	0.073
	Positive	3 (42.9)	7 (12.5)	
***TCRA/D* translocations**	Negative	5 (71.4)	38 (67.9)	0.848
	Positive	2 (28.6)	18 (32.1)	
***TCRA/D* deletions**	Negative	6 (85.7)	54 (96.4)	0.302
	Positive	1 (14.3)	2 (3.6)	
***TCRA/D* amplifications**	Negative	3 (42.9)	49 (87.5)	**0.014**
	Positive	4 (57.1)	7 (12.5)	
***Total TCRA/D* abnormalities**	Negative	1 (14.3)	32 (57.1)	**0.047**
	Positive	6 (85.7)	24(42.9)	
***TCRβ***	Negative	5 (71.4)	45 (80.4)	0.627
	Positive	2 (28.6)	11 (19.6)	
**MRD15**	< 0.01	0 (0)	10 (21.7)	0.363
	≥ 0.01	3 (100)	33 (78.3)	
	< 0.1	0 (0)	16 (37.2)	0.542
	≥ 0.1	3 (100)	27 (62.8)	
**MRD42**	< 0.01	1 (25.0)	14 (36.8)	0.638
	≥ 0.01	3 (75.0)	24 (63.2)	
	< 0.1	2 (50)	27 (71.1)	0.576
	≥ 0.1	2 (50)	11 (28.9)	
**Relapse**	Negative	6 (85.7%)	49 (87.5%)	0.894
	Positive	1 (14.3%)	7 (12.5%)	
**Early death**	Negative	5 (71.4%)	47 (83.9%)	0.595
	Positive	2 (28.6%)	9 (16.1%)	
**Death**	Negative	2 (28.6%)	34 (60.7%)	0.128
	Positive	5 (71.4%)	22 (39.3%)	

Data are presented as number (percentage) or median and range

*BM* bone marrow, *BP* peripheral blood, *CD* cluster of differentiation, *CR* complete remission, *IPT* immunophenotyping, *MCN* modal chromosomal number, *MRD* minimal residual disease, *TCR* T-cell receptors, *TLC* total leukocyte count, *WBC* white blood cells count

**Fig. 2:**
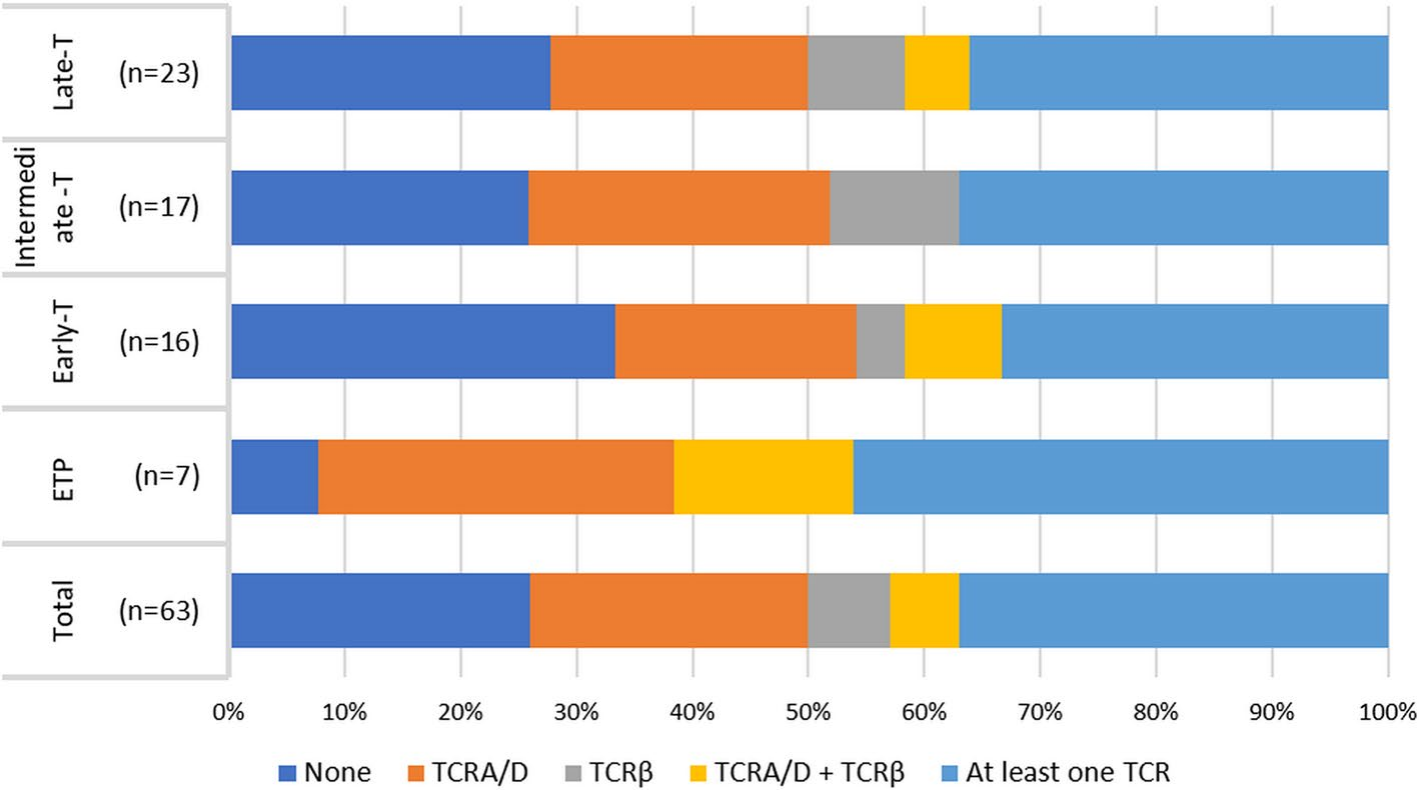
Frequency of TCR aberrations at the initial diagnosis according to the immunophenotype subgroups of T-ALL patients. Early-T phenotypes are double negative for CD4 and CD8; intermediate-T phenotypes were double positive for CD4 and CD8. Late T-phenotype: single positive for either CD4 or CD8

Interestingly, there was 85.7% (6/7) of the ETP-ALL patients showed increased incidence of total *TCRA/D* abnormalities compared to 42.9% (24/56) in non-ETP patients, *P* = *0.047*. Similarly, there was a significant association between ETP-ALL and *TCRA/D* amplifications (*P* = *0.014*), as *TCRA/D* amplifications were present in 57.1% (4/7) of ETP patients compared to 12.5% (7/56) in non-ETP patients (Fig. 2).

### Association between TCR aberrations and ALL patients’ characteristics

Among patients presenting with bleeding, 71.4% were positive to *TCRA/D* translocations (*P* = *0.028*). Patients with *TCRA/D* amplification had lower WBC count (median-range): 52 (3–546) vs 240 (2–967), *P* = *0.02*), lower percentage of BM blast cells (median-range): 80 (38–92) vs 90 (0–99), *P* = *0.05*), compared to *TCRA/D* amplification-negative cases. Additionally, patients with *TCRA/D* translocations were positively associated with aberrant myeloid markers (*P* = *0.06*); had additional chromosomal abnormalities (54.5% vs 23.1%, *P* = *0.036*); and, subsequently, presented in complex karyotypes (45.5% vs 9.6%, *P* = *0.003*) compared to those who did not show *TCRA/D* amplification (Table 3).

**Table 3:** Clinical features, laboratory characteristics, and outcome in relation to *TCRA/D* translocation and *TCRA/D* amplification

Parameters		*TCRA/D* translocation		*p*-value	*TCRA/D* amplification		*p*-value
		**Negative**	**Positive**		**Negative**	**Positive**	
Age	Median (range)	7 (2–18)	6 (1–18)	0.841	6.5 (1–18)	5 (3–17)	0.669
Hemoglobin (g/dl)	Median (range)	7.4 (4–15)	8.4 (5–14)	0.460	7.8 (4–14)	7.4 (6–15)	0.625
Platelets’ count (× 10^9^/L)	Median (range)	55 (8–693)	43 (17–223)	0.194	49 (9–693)	44 (8–224)	0.779
WBC (× 10^9^/L)	Median (range)	160 (2–967)	223 (4–746)	0.988	240 (2–967)	52 (3–546)	0.020
PB blast%	Median (range)	80 (0–99)	85 (0–97)	0.529	85 (0–99)	50 (0–95)	0.154
BM blast%	Median (range)	89 (0–99)	90 (28–98)	0.784	90 (0–99)	80 (38–92)	0.050
Sex	Male	29 (67.4%)	15 (75.0%)	0.543	35 (67.3%)	9 (81.8%)	0.341
	Female	14 (32.6%)	5 (25.0%)		17 (32.7%)	2 (18.2%)	
BM cellularity	Hypercellular	34 (79.1%)	17 (85%)	0.737	44 (84.6%)	7 (63.6%)	0.197
	Normocellular	9 (20.9%)	3 (15%)		8 (15.4%)	4 (36.4%)	
CD34	Negative	38 (88.4%)	15 (75%)	0.266	43 (82.7%)	10 (90.9%)	0.676
	Positive	5 (11.6%)	5 (25%)		9 (17.3%)	1 (9.1%)	
ETP	Negative	38 (88.4%)	18 (90%)	0.848	49 (94.2%)	7 (63.6%)	0.014
	Positive	5 (11.6%)	2 (10%)		3 (5.8%)	4 (36.4%)	
MCN	Normal	30 (69.8%)	16 (80.0%)	0.629	43 (82.7%)	3 (27.3%)	*P* < 0.001
	Hypodiploidy	5 (11.6%)	1 (5.0%)		6 (11.5%)	0 (0.0%)	
	Hyperdiploidy	8 (18.6%)	3 (15.0%)		3 (5.8%)	8 (72.7%)	
TCR_B	Negative	32 (74.4%)	18 (90.0%)	0.155	44 (84.6%)	6 (54.5%)	0.025
	Positive	11 (25.6%)	2 (10.0%)		8 (15.4%)	5 (45.5%)	
Complex (presence of 3 or more cytogenetics abnormalities)	Negative	35 (81.4%)	18 (90.0%)	0.384	47 (90.4%)	6 (54.5%)	0.003
	Positive	8 (18.6%)	2 (10.0%)		5 (9.6%)	5 (45.5%)	
MRD15	< 0.01	6 (19.4%)	3 (23.1%)	0.780	9 (23.1%)	0 (0.0%)	0.566
	≥ 0.01	25 (80.6%)	10 (76.9%)		30 (76.9%)	5 (100%)	
MRD42	< 0.01	9 (34.6%)	6 (37.5%)	0.850	14 (37.8%)	1 (20.0%)	0.639
	≥ 0.01	17 (65.4%)	10 (62.5%)		23 (62.2%)	4 (80.0%)	
Relapse	Negative	36 (83.7%)	19 (95%)	0.418	44 (84.6%)	11 (100%)	0.331
	Positive	7 (16.3%)	1 (5%)		8 (15.4%)	0 (0.0%)	
Early death	Negative	35 (81.4%)	17 (85%)	0.726	44 (84.6%)	8 (72.7%)	0.389
	Positive	8 (18.6%)	3 (15%)		8 (15.4%)	3 (27.3%)	
Death	Negative	21 (48.8%)	15 (75%)	0.061	31 (59.6%)	5 (45.5%)	0.507
	Positive	22 (51.2%)	5 (25%)		21 (40.4%)	6 (54.5%)	

Data are presented as number (percentage) or median and range

*BM* bone marrow, *BP* peripheral blood, *CD* cluster of differentiation, *CR* complete remission, *MCN* modal chromosomal number, *MRD* minimal residual disease, *TCR* T-cell receptors, *TLC* total leukocyte count, *WBC* white blood cells count

Notably, there was a highly statistically significant relation between *TCR-*copy number variation groups (CNV) and MCN, where positive cases to *TCRA/D* amplification had higher MCN with a median of 50 (range: 46–91) chromosomes (*P* < *0.01*). While all cases with *TCRA/D* deletion cases had MCN lower than 46 with a median of 45 (range: 41–45) chromosomes (*P* < *0.001,* supp. 2). There was a significant association between *TCRA/D*-abnormalities and hyperdiploidy, as 10/11 (90.9%) of the patients with hyperdiploidy showed *TCRA/D* abnormalities (*P* = *0.006*).

On the other hand, patients with *TCR-β* aberrations had lower WBC count (median-range)*:*75 (2–883) vs 269 (2–967), *P* = *0.002*), lower risk of CNS involvement at presentation (53.8% vs 94%, *P* = *0.003*), positive association with CD7 expression (84.6% vs 51%, *P* = *0.05*) compared to the ALL patients without *TCR-β* aberrations (Table 3).

### Impact of ETP and TCR aberrations on the T-ALL patients’ response to treatment

Minimal residual disease (MRD) was performed by flow cytometry for 46 patients on day 15, and for 42 patients on day 42 according to the adequacy of the samples and the presence of leukemia-associated immunophenotypes (Table 1).

Patients with ETP-ALL showed a nonsignificant inferior clearance rate of leukemic cells compared to the other T-ALL patients, as all patients had detectable MRD (≥ 0.01%) after 15 days of treatment (Table 2). Only two patients had *MRD* ≥ 1% at the end of induction of whom one patient had ETP-ALL. However, due to the low number of patients, no statistical analysis could be done.

Eleven patients died during induction, 7 were from treatment-related causes (TRM), while 4 were from disease-related causes. Two of the seven patients with ETP (28.6%) died in induction from disease-related cause, while 9/56 patients with non-ETP (16%) died mainly due to TRM.

**Fig. 3:**
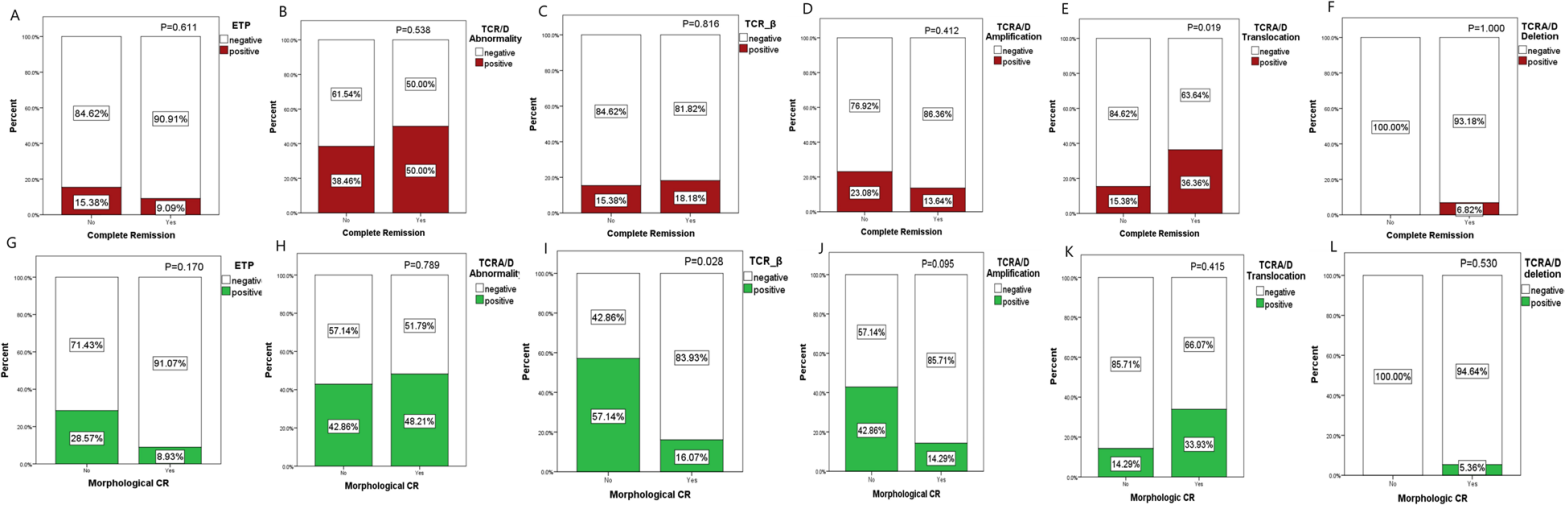
**A**–**F** showing the association of ETP and TCR abnormalities with the patients’ complete remission, while **G**–**L** showing the association of ETP and TCR abnormalities with the patients’ morphological complete remission. The colored parts represent the percent of patients who were positive for the ETP or TCR abnormalities

The morphological CR of the assessed ALL patients was significantly associated with the presence of *TCR-β* aberrations (*P* = *0.028*), while a nonsignificant association between *TCRA/D* amplification and the achievement of the morphological CR was noted (*P* = *0.095,* Fig. 3). In addition, patients with *TCR-β* abnormalities were more likely to have negative MRD on day 42 compared to *TCR-β*-negative patients (80% vs 29.7%, *P* = *0.047*, Tables 3 and 4).

**Table 4:** Clinical features, laboratory characteristics, and outcome in relation to total *TCR-B* aberrations and *TCRA/D* abnormality

Parameters		*TCR-B* aberrations		*p-value*	*TCRA/D* abnormality		*p-value*
		Negative	Positive		Negative	Positive	
Age	Median (range)	6 (1–18)	6.5 (3–17)	0.676	6 (2–18)	6.5 (1–18)	0.629
Hemoglobin (g/dl)	Median (range)	7.6 (4–15)	8 (5–11)	0.760	7.2 (4–12)	8.4 (5–14.5)	0.338
Platelets’ count (× 109/L)	Median (range)	48 (8–693)	48 (9–224)	0.508	50 (9–693)	44.5 (8–224)	0.429
WBC (× 109/L)	Median (range)	269 (2–967)	75 (2–883)	0.022	230 (2–967)	144 (3–746)	0.177
PB blast%	Median (range)	83 (0–99)	80 (0–99)	0.349	80 (0–99)	83 (0–97)	0.847
BM blast%	Median (range)	90 (0–99)	85 (20–97)	0.104	90 (0–99)	88.5 (28–98)	0.238
Sex	Male	35 (70.0%)	9 (69.2%)	0.957	20 (60.6%)	24 (80.0%)	0.094
	Female	15 (30.0%)	4 (30.8%)		13 (39.4%)	6 (20.0%)	
Initial CNS	CNSI	37 (74%)	11 (84.6%)	0.003	4 (12.1%)	5 (16.7%)	0.346
					23 (69.7%)	16 (53.3%)	
	TLP	11 (22%)	2 (15.4%)		6 (18.2%)	7 (23.3%)	
	CNSIII	2 (4%)	0 (0.0%)		0 (0.0%)	2 (6.7%)	
BM cellularity	Hypercellular	40 (80%)	11 (84.6%)	0.706	27 (81.8%)	24 (80.0%)	0.845
	Normocellular	10 (20%)	2 (15.4%)		6 (18.2%)	6 (20.0%)	
CD34	Negative	40 (80%)	13 (100%)	0.105	30 (90.9%)	23 (76.7%)	0.172
	Positive	10 (20%)	0 (0.0%)		3 (9.1%)	7 (23.3%)	
ETP	Negative	45 (90%)	11 (84.6%)	0.627	32 (97.0%)	24 (80.0%)	0.047
	Positive	5 (10%)	2 (15.4%)		1 (3.0%)	6 (20.0%)	
MCN	Normal	39 (78.0%)	7 (53.8%)	0.081	29 (87.9%)	17 (56.7%)	0.006
	Hypodiploidy	5 (10.0%)	1 (7.7%)		3 (9.1%)	3 (10.0%)	
	Hyperdiploidy	6 (12.0%)	5 (38.5%)		1 (3.0%)	10 (33.3%)	
Other chromosomal abnormalities	Negative	40 (80.0%)	5 (38.5%)	0.003	25 (75.8%)	20 (66.7%)	0.425
	Positive	10 (20%)	8 (61.5%)		8 (24.2%)	10 (33.3%)	
Complex	Negative	44 (88.0%)	9 (69.2%)	0.099	29 (87.9%)	24 (80.0%)	0.393
	Positive	6 (12.0%)	4 (30.8%)		4 (12.1%)	6 (20.0%)	
MRD15	< 0.01	6 (17.1%)	3 (33.3%)	0.360	6 (23.1%)	3 (16.7%)	0.716
	≥ 0.01	29 (82.9%)	6 (66.7%)		20 (76.9%)	15 (83.3%)	
MRD42	< 0.01	11 (29.7%)	4 (80.0%)	0.047	9 (42.9%)	6 (28.6%)	0.520
	≥ 0.01	26 (70.3%)	1 (20.0%)		12 (57.1%)	15 (71.4%)	
Relapse	Negative	42 (84.0%)	13 (100%)	0.188	27 (81.8%)	28 (93.3%)	0.261
	Positive	8 (16.0%)	0 (0.0%)		6 (18.2%)	2 (6.7%)	
Death	Negative	30 (60.0%)	6 (46.2%)	0.531	17 (51.5%)	19 (63.3%)	0.446
	Positive	20 (40.0%)	7 (53.8%)		16 (48.5%)	11 (36.7%)	
Early death	Negative	44 (88.0%)	8 (61.5%)	0.040	27 (81.8%)	25 (83.3%)	0.478
	Positive	6 (12.0%)	5 (38.5%)		6 (18.2%)	5 (16.7%)	

Data are presented as number (percentage) or median and range

*BM* bone marrow, *BP* peripheral blood, *CD* cluster of differentiation, *CNS* central nervous system, *CR* complete remission, *MCN* modal chromosomal number, *MRD* minimal residual disease, *TCR* T-cell receptors, *TLC* total leukocyte count, *TLP* traumatic lumbar puncture, *WBC* white blood cells count

### Impact of ETP and TCR aberrations on the clinical outcome of the assessed ALL patients

With a median follow-up of 22 (range: 1–86) months, eleven patients (17.5%) died during the induction, 8 cases (12.7%) relapsed after achieving CR of which 1 (14.3%), and 2 (64.2%) patients were positive to ETP and *TCR* abnormalities, respectively. The total number of deaths at the end of the study was 27 cases (42.9%). Characteristics of the relapsed pediatric T-ALL patients are illustrated in supp. 3.

**Fig. 4:**
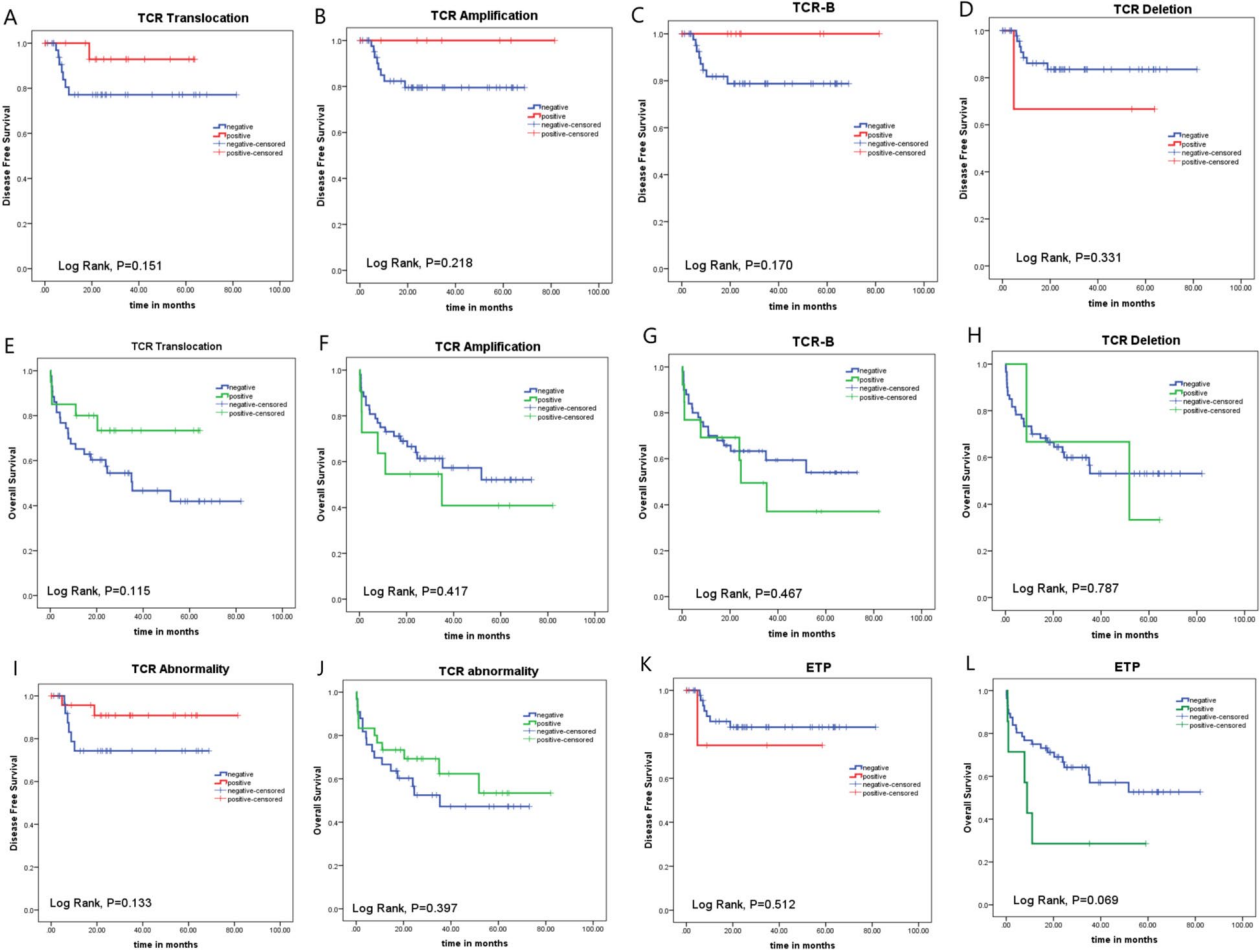
Survival of pediatric T-ALL according to TCR aberrations status and ETP. **A**, **B**, **C**, **D**, **I**, and **K** Disease-free survival (DFS) and **E**, **F**, **G**, **H**, **J**, and **L** overall survival (OS) of different TCR aberrations

Notably, 5/13 (38.5%) of patients with *TCR-β* aberrations died before the end of the induction compared to 6/50 (12%) of the negative cases (*P* = *0.04*). The present study showed that there was no significant impact of *TCR* aberrations or ETP on the DFS or the OS of the assessed T-ALL patients (*P* > *0.05,* Fig. 4).

## Discussion

Acute lymphoblastic leukemia is the most common childhood malignancy. Although first described in 2009 by Coustan-Smith et al. [19], ETP-ALL was included, as a provisional entity only in the 2016 revision of the “WHO classification of tumors of hematopoietic and lymphoid tissues” [20] recognizing its fascinating scenario of biological heterogeneity [4]. Nevertheless, the distinction of ETP-ALL is still challenging. The “partial CD5 expression” criterion had a negative impact on the correct identification of ETP-ALL cases, as ETP patients with high CD5 expression showed a comparable survival to non-ETP T-ALL. Consequently, Zuubier et al. [21] proposed refined immunophenotypic criteria by excluding CD5 expression and adding negativity for CD4, categorizing the cases with elevated CD5 expression as “near ETP” [4, 22, 23].

In the current series, we analyzed the frequency, clinical, and prognostic features of pediatric ETP and *TCRA/D*-ALL-positive cases compared to the other T-ALL cases. Based on the immunophenotypic criteria proposed by recent studies [5, 6, 22–25], our data showed that ETP-ALL represented 11.1% of T-ALL patients which goes in agreement with the previously reported frequency in pediatric T-ALL [18, 21–23]. In contrast, a higher frequency (17%) was detected by Burns et al. [1], which was comparable to that observed in adult patients, due to the inclusion of patients ranged from 1 to 21 years in their series compared to 1–18 years in our cohort.

In concordance with previous studies [1, 5, 15, 19, 21–23, 26], our series demonstrated that there were no significant differences between the ETP and non-ETP patients except that ETP patients were older, presented with lower WBC count, and had lower percentage of PB blast cells than non-ETP patients. In contrast to these findings, Jain et al. [24] reported higher BM blast count and CNS involvement at presentation, while Puglianini et al. [6] reported an association of ETP with younger age. Moreover, several studies [5, 6, 21, 24] reported the frequent occurrence of ETP in males compared to female patients.

In the present study, most of our ETP-ALL patients (85.7%) had chromosomal aberrations and were less likely to have diploid karyotype (28.6%). In addition, ETP-ALL patients had a significant association with hyperdiploid karyotype, *TCRA/D* gene amplification, and a nonsignificant trend to have complex karyotypes. These findings were consistent with the other published series [4, 6, 19, 22, 24, 25], which reported that the higher likelihood of ETP-ALL patients harboring clonal cytogenetic lesions, with highly variable non-diploid karyotypes, and lower frequency of classical T-ALL translocations than the other T-ALL patients. In the same context, Coustan-Smith et al. [19] reported that ETP-ALL patients had significantly more DNA copy number abnormalities (genomic gains or losses) than other T-ALL patients. This high genomic instability may provide an explanation for the higher incidence of hyperdiploid karyotype and *TCRA/D* gene amplification in ETP phenotype compared to non-ETP patients in our series.

There was a nonsignificant association between ETP-ALL patients and positive MRD (> 0.1) after induction therapy; in addition, it was noted that all patients with ETP-ALL had detectable MRD on day 15. This goes in agreement with previous studies [1, 5, 6, 19, 22–24], which reported a lower incidence of clearance of leukemic cells after the first phase of induction therapy in patients with ETP-ALL compared to other T-ALL.

In line with Morita et al. [22], our study showed that patients with ETP-ALL tend to have worse OS than non-ETP patients, with no significant impact on patients’ DFS. In concordance with Wood et al. [23], no relapses occurred in patients with ETP-ALL later than 12-month post-diagnosis.

There was a discrepancy in long-term outcomes of ETP-ALL patients in different studies compared to non-ETP patients. The original early studies [1, 6, 19] reported that patients with ETP-ALL had a significantly worse OS and DFS, while the recent studies [4, 5, 23, 25–27] reported that ETP-ALL was not associated with a poor outcome, despite the higher incidence of a positive MRD at the end of induction. Such discrepancy may be explained by the limited numbers of pediatric patients enrolled in the early studies, while the recent studies were based on larger number of children. In addition, this was also likely due to the treatment intensification of the MRD-positive patients that abolished the negative prognostic impact of ETP-ALL [23].

In line with the previous studies [19], the frequency of *TCRA/D* aberrations in pediatric patients with T-ALL was 47.6% upon screening by FISH, which was higher than that previously reported by Kim et al. [28].

In agreement with the other series [8, 9], the incidence of TCR rearrangements was 20%. Interestingly, patients with *TCRA/D* amplification tend to have lower WBC count and lower percentage of BM blast cells and were positively associated with aberrant myeloid markers*.* In addition, patients with *TCRA/D* amplification had hyperdiploid karyotype and presented in complex karyotypes when compared to *TCRA/D* amplification-negative patients. These associations are similar to that found in patients with ETP-ALL which may explain the strong association of *TCRA/D* amplification in patients with ETP-ALL. In line with Kim et al. [28] who detected two or more instances of TCR clonality in most children with T-ALL, patients with *TCRA/D* amplification had concurrent *TCR-β* aberrations.

Moreover, the current study demonstrated that patients with *TCR-β* aberrations showed a morphological CR as well as negative MRD on day 42 compared to *TCR-β*-negative patients. These data are in agreement with Dutta et al. [29] who concluded a better outcome in patients with TCR-*β* selection. Also, Brüggemann et al. [30] found that clonal TCR-*β* gene rearrangements are excellent targets for RQ-PCR detection of MRD in T-ALL patients.

High percentage of pediatric and young adult patients achieves early MRD negativity; more than 80% of T-ALL patients remain MRD positive at the end of induction as demonstrated in the AIEOP-BFM-ALL 2000 study [31].

In agreement with previously published papers [8, 9], the present study showed that cytogenetic features do not play a prognostic role in T-ALL, nor do types of TCR aberrations as there was no significant difference between *TCRA/D* aberrations positive and negative cases regarding the DFS or OS.

## Conclusion

In summary, there was a nonsignificant trend of ETP-positive cases to have lower outcome but mainly due to increased mortalities. *TCR-β* aberrations were significantly associated with negative MRD at the end of induction compared to *TCR-β*-negative patients. No significant association was found between TCR aberrations and DFS and OS. However, the main limitation of the present study was the small number of the ETP-ALL-positive patients in relation to the other compared non-ETP-ALL patients which affects the reliability of the results. Therefore, further studies with a larger number of patients are highly required for better validation of the data. This will allow for proper integrating the genetic and the clinico-biological data of the T-ALL patients, translating them into better risk stratification, and subsequently improving the outcomes of T-ALL patients.

## Supplementary Information

**Additional file 1: Supp. 1.** St. Jude total XV protocol for treatment of newly diagnosed patients with Acute Lymphoblastic Leukemia.

**Additional file 2: Supp. 2.** Clinical features, laboratory characteristics and outcome in relation to TCRA/D deletion*.*

**Additional file 3: Supp. 3.** Clinical characteristics of relapsed pediatric T-ALL patients.

## Data Availability

Data supporting the findings are included in the manuscript, and any additional data are available at the corresponding author on request.

## Abbreviations

BM: Bone marrow
CD: Cluster of differentiation
CNV: Copy number variation groups
CSF: Cerebrospinal fluid
DFS: Disease-free survival
ETP: Early T-cell precursor
FISH: Fluorescent in situ hybridization
HSCT: Hematopoietic stem cell therapy
IQR: Interquartile ranges
MCN: Median of modal chromosome number
MRD: Minimal residual disease
NCI: National Cancer Institute
OS: Overall survival
PB: Peripheral blood
T-ALL: T-cell acute lymphoblastic leukemia
TCR: T-cell receptors

## References

[1] Burns MA, Place AE, Gutiérrez A, Forrest S, Stevenson K, et al. Identification of prognostic factors in childhood T-cell acute lymphoblastic leukemia: results from DFCI ALL Consortium Protocols 05–001 and 11–001. Pediatr Blood Cancer. 2021;68: e28719. 33026184 10.1002/pbc.28719 PMC8369809

[2] Raimondi S. T-lineage acute lymphoblastic leukemia (T-ALL). Atlas Genet Cytogenet Oncol Haematol. 2007;11:328–39.

[3] Girardi T, Vicente C, Cools J, De Keersmaecker K. The genetics and molecular biology of T-ALL. Blood. 2017;129(9):1113–23. 10.1182/blood-2016-10-706465. 28115373 10.1182/blood-2016-10-706465 PMC5363819

[4] Tarantini F, Cumbo C, Anelli L, et al. (2021): Inside the biology of early T-cell precursor acute lymphoblastic leukemia: the perfect trick. Biomarker Res. 2021;9:89. 10.1186/s40364-021-00347-z PMC8686563 34930475

[5] Genescà E., Morgades M., Montesinos P., Barba P., et al. Unique clinico-biological, genetic and prognostic features of adult early T-cell precursor acute lymphoblastic leukemia. Haematologica. 2020;105:e294. 10.3324/haematol.2019.225078 PMC7271589 31537688

[6] Puglianini O, Papadantonakis N. Early precursor T-cell acute lymphoblastic leukemia: current paradigms and evolving concepts. Ther Adv Hematol. 2020;11:1–13. 10.1177/2040620720929475 PMC7370557 32733662

[7] Aifantis I, Raetz E, Buonamici S. Molecular pathogenesis of T-cell leukaemia and lymphoma. Nat Rev Immunol. 2008;8(5):380–90. 10.1038/nri2304. 18421304 10.1038/nri2304

[8] Karrman K, Forestier E, Heyman M, et al. Clinical and cytogenetic features of a population-based consecutive series of 285 pediatric T-cell acute lymphoblastic leukemias: rare T-cell receptor gene rearrangements are associated with poor outcome. Genes chromosomes Cancer. 2009;48(9):795–805. 10.1002/gcc.20684. 19530250 10.1002/gcc.20684

[9] Karrman K, Johansson B. Pediatric T-cell acute lymphoblastic leukemia. Genes Chromosom Cancer. 2017;56(2):89–116. 10.1002/gcc.22416. 27636224 10.1002/gcc.22416

[10] Bond J, Bergon A, Durand A, Tigaud I, et al. Cryptic XPO1-MLLT10 translocation is associated with HOXA locus deregulation in T-ALL. Blood. 2014;124:3023–5. 25377562 10.1182/blood-2014-04-567636 PMC4224197

[11] Hunger SP, Lu X, Devidas M, et al. Improved survival for children and adolescents with acute lymphoblastic leukemia between 1990 and 2005: a report from the children’s oncology group. J Clin Oncol. 2012;30(14):1663–9. 22412151 10.1200/JCO.2011.37.8018 PMC3383113

[12] Hoed DM, Pluijm SMF, te Winkel ML, et al. Aggravated bone density decline following symptomatic osteonecrosis in children with acute lymphoblastic leukemia. Haematologica. 2015;100(12):1564–70. 26405155 10.3324/haematol.2015.125583 PMC4666332

[13] Litzow MR. How we treat T-cell acute lymphoblastic leukemia in adults. Blood. 2015;126(7):833–41. 25966987 10.1182/blood-2014-10-551895

[14] Bhojwani D, Pui C-H. Relapsed childhood acute lymphoblastic leukaemia. Lancet Oncol. 2013;14(6):e205–17. 23639321 10.1016/S1470-2045(12)70580-6

[15] McGowan-Jordan J, Hastings R, Moore S.. Re: International System for Human Cytogenetic or Cytogenomic Nomenclature (ISCN): Some Thoughts, by T. Liehr. Cytogenet Genome Res. 2021;161(5):225–6. 10.1159/000516655. 10.1159/000516655 34407535

[16] McGowan-Jordan J, Hastings Ros J. and Moore S. ISCN 2020: An International System for Human Cytogenomic Nomenclature. 2020. 10.1159/isbn.978-3-318-06867-2.

[17] Pui CH, Relling MV, Sandlund JT, Downing JR, Campana D, Evans WE. Rationale and design of Total Therapy Study XV for newly diagnosed childhood acute lymphoblastic leukemia. Ann Hematol. 2004;83 Suppl 1:S124–6. 10.1007/s00277-004-0850-2. 10.1007/s00277-004-0850-2 15124703

[18] Pui CH, Campana D, Pei D et al. Treatment of childhood acute lymphoblastic leukemia without prophylactic cranial irradiation. N Engl J Med. 2009;360(26): 2730–41. 10.1056/NEJMoa0900386. 10.1056/NEJMoa0900386 PMC2754320 19553647

[19] Coustan-Smith E, Mullighan CG, Mihaela OM, et al. Early t-cell precursor leukemia: a subtype of very high-risk acute lymphoblastic leukemia identified in two independent cohorts. Lancet oncol. 2009;10(2):147–56. 10.1016/s1470-2045(08)70314-0. 19147408 10.1016/S1470-2045(08)70314-0 PMC2840241

[20] Arber DA, Orazi A, Hasserjian R, et al. The 2016 revision to the World Health Organization classification of myeloid neoplasms and acute leukemia. Blood. 2016;127(20):2391–405. 27069254 10.1182/blood-2016-03-643544

[21] Zuurbier L., Gutierrez A., Mullighan C., et al. Immature MEF2C-dysregulated T-cell leukemia patients have an early T-cell precursor acute lymphoblastic leukemia gene signature and typically have non-rearranged T-cell receptors. Haematologica. 2014;99(1):94–102. 10.3324/haematol.2013.090233 PMC4007923 23975177

[22] Morita K, Jain N, Kantarjian H, et al. Outcome of T-cell acute lymphoblastic leukemia/lymphoma: focus on near-ETP phenotype and differential impact of nelarabine. Am J Hematol. 2021;96:589–98. 33639000 10.1002/ajh.26144

[23] Wood BL, Winter SS, Dunsmore KP, Devidas M, et al. T-lymphoblastic leukemia (T-ALL) shows excellent outcome, lack of significance of the early thymic precursor (ETP) immunophenotype, and validation of the prognostic value of end-induction minimal residual disease (MRD) in Children’s Oncology Group (COG) study AALL0434. Blood. 2014;124(21):1.24993873

[24] Jain N, Lamb AV, O’Brien S. Early T-cell precursor acute lymphoblastic leukemia/lymphoma (ETP-ALL/LBL) in adolescents and adults: a high-risk subtype. Blood. 2016;127(15):1863–9. 26747249 10.1182/blood-2015-08-661702 PMC4915808

[25] Patrick K, Wade R, Goulden N, Mitchell C, et al. Outcome for children and young people with Early T-cellprecursor acute lymphoblastic leukaemia treated on acontemporary protocol, UKALL 2003. Br J Haematol. 2014;166:421–4. 24708207 10.1111/bjh.12882

[26] Noronha EP, Marques LV, Andrade FG, et al. The profile of immunophenotype and genetic aberrations in subsets of pediatric T-acute lymphoblastic leukemia. Front Oncol. 2019;9:316–25. 31338319 10.3389/fonc.2019.00316 PMC6503680

[27] Shiraz P, Jehangir W, Agrawal V. T-cell acute lymphoblastic leukemia current concepts in molecular biology and management. Biomedicines. 2021;9(11):1621. 34829849 10.3390/biomedicines9111621 PMC8615775

[28] Kim H, Kim I, Chang C, Kong SY, et al. T-cell receptor rearrangements determined using fragment analysis in patients with T-acute lymphoblastic leukemia. Ann Lab Med. 2019;39:125–32. 30430774 10.3343/alm.2019.39.2.125 PMC6240512

[29] Dutta A, Zhao B, Love PE. New insights into TCR β-selection. Trends Immunol. 2021;42(8):735–50. 10.1016/j.it.2021.06.005. 34261578 10.1016/j.it.2021.06.005

[30] Brüggemann M, van der Velden VH, Raff T, Droese J, Ritgen M, et al. Rearranged T-cell receptor beta genes represent powerful targets for quantification of minimal residual disease in childhood and adult T-cell acute lymphoblastic leukemia. Leukemia. 2004;18(4):709–19. 10.1038/sj.leu.2403263. 14961040 10.1038/sj.leu.2403263

[31] Chen YL, Su IJ, Cheng HY, et al. BIOMED-2 protocols to detect clonal immunoglobulin and T-cell receptor gene rearrangements in B- and T-cell lymphomas in southern Taiwan. Leuk Lymphoma. 2010;51(4):650–5. 20233058 10.3109/10428191003660631

