# Supplementary figures and images for: Clinical features, laboratory characteristics, and outcome of ETP and TCRA/D aberrations in pediatric patients with T-acute lymphoblastic leukemia
Source: J Egypt Natl Canc Inst. 2023 Jun 12;35:17. doi: 10.1186/s43046-023-00176-1 (PMC13313959; doi:10.1186/s43046-023-00176-1)

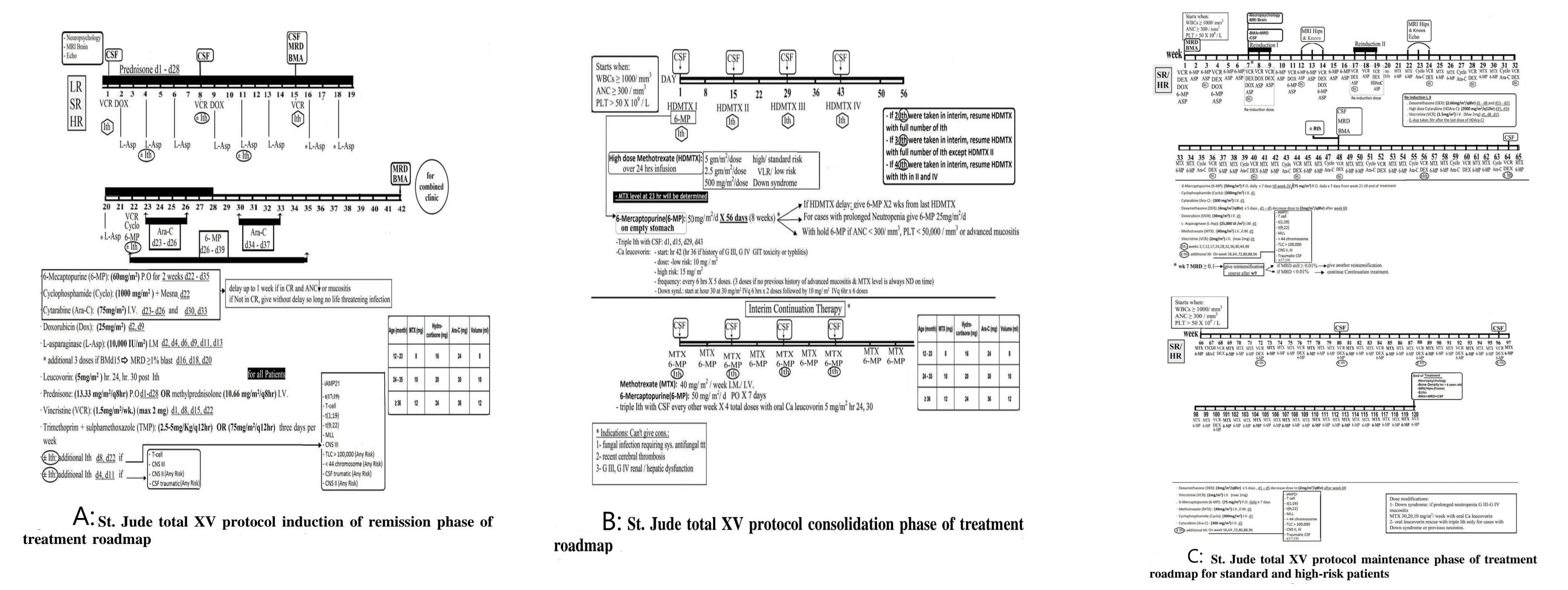

Supplement: Supplementary file 1 — Additional file 1: Supp. 1. St. Jude total XV protocol for treatment of newly diagnosed patients with Acute Lymphoblastic Leukemia. [file 43046_2023_176_MOESM1_ESM.jpg]
