# Supplementary material for: Clinical features, laboratory characteristics, and outcome of ETP and TCRA/D aberrations in pediatric patients with T-acute lymphoblastic leukemia
Source: J Egypt Natl Canc Inst. 2023 Jun 12;35:17. doi: 10.1186/s43046-023-00176-1 (PMC13313959; doi:10.1186/s43046-023-00176-1)
Supplement: Supplementary file 2 — Additional file 2: Supp. 2. Clinical features, laboratory characteristics and outcome in relation to TCRA/D deletion. [file 43046_2023_176_MOESM2_ESM.docx]

**Supp. 2:** Clinical features, laboratory characteristics and outcome in relation to TCRA/D deletion

| **Patients’ characteristics** | | ***TCRA/D deletion*** | | ***P value*** |
| --- | --- | --- | --- | --- |
|  |  | negative | positive |  |
| Age | Median (IQR) | 6 (1-18) | 13 (6-14) | 0.231 |
| Haemoglobin (g/dl) | Median (IQR) | 7.8 (4-14.5) | 7.6 (7-8.7) | 0.867 |
| Platelets’ count (x109/L) | Median (IQR) | 48.5 (8-693) | 43 (26-106) | 0.796 |
| WBC (x109/L) | Median (IQR) | 180 (2-967) | 258 (100-659) | 0.570 |
| PB blast% | Median (IQR) | 81 (0-99) | 95 (30-97) | 0.411 |
| BM blast% | Median (IQR) | 89 (0-99) | 88 (88-90) | 0.867 |
| Sex | Male | 41 (68.3%) | 3 (100%) | 0.243 |
|  | Female | 19 (31.7%) | 0 (0.0%) |  |
| Hepatomegaly | negative | 9 (15%) | 1 (33.3%) | 0.410 |
|  | positive | 51 (85%) | 2 (66.7%) |  |
| Splenomegaly | negative | 11 (18.3%) | 1 (33.3%) | 0.76 |
|  | positive | 49 (81.7%) | 2 (66.7%) |  |
| LN | negative | 5 (8.3%) | 0 (0.0%) | 0.602 |
|  | positive | 55 (91.7%) | 3 (100%) |  |
| Mediastinal Mass | negative | 35 (58.3%) | 1 (33.3%) | 0.572 |
|  | positive | 25 (41.7%) | 2 (66.7%) |  |
| Initial CNS | negative | 9 (15.0%) | 0 (0.0%) | 0.848 |
|  | CNSI | 37 (61.7%) | 2 (66.7%) |  |
|  | TLP | 12 (20.0%) | 1 (33.3%) |  |
|  | CNSIII | 2 (3.3%) | 0 (0.0%) |  |
| CSF infiltration | negative | 59 (98.3%) | 3 (100%) | 0.822 |
|  | positive | 1 (1.7%) | 0 (0.0%) |  |
| Fever | negative | 22 (36.7%) | 0 (0.0%) | 0.546 |
|  | positive | 38 (63.3%) | 3 (100%) |  |
| Anemia | negative | 24 (40.0%) | 1 (33.3%) | 0.818 |
|  | positive | 36 (60.0%) | 2 (66.7%) |  |
| Bleeding | negative | 53 (88.3%) | 3 (100%) | 0.530 |
|  | positive | 7 (11.7%) | 0 (0.0%) |  |
| BM Cellularity | Hypercellular | 48 (80.0%) | 3 (100%) | 0.389 |
|  | Normocellular | 12 (20.0%) | 0 (0.0%) |  |
| IPT diagnosis | T intermediate | 16 (26.7%) | 1 (33.3%) | 0.444 |
|  | T early | 15 (25.0%) | 1 (33.3%) |  |
|  | T late | 23 (38.3%) | 0 (0.0%) |  |
|  | ETP | 6 (10.0%) | 1 (33.3%) |  |
| CD34 | negative | 51 (85.0%) | 2 (66.7%) | 0.410 |
|  | positive | 9 (15.0%) | 1 (33.3%) |  |
| ETP | negative | 54 (90%) | 2 (66.7%) | 0.302 |
|  | positive | 6 (10%) | 1 (33.3%) |  |
| CD3 surface | negative | 51 (85.0%) | 2 (66.7%) | 0.410 |
|  | positive | 9 (15.0%) | 1 (33.3%) |  |
| CD2 | negative | 31 (51.7%) | 2 (66.7%) | 0.902 |
|  | positive | 29 (48.3%) | 1 (33.3%) |  |
| CD5 | negative | 42 (70.0%) | 2 (66.7%) | 0.902 |
|  | positive | 18 (30.0%) | 1 (33.3%) |  |
| CD7 | negative | 24 (40.0%) | 2 (66.7%) | 0.564 |
|  | positive | 36 (60.0%) | 1 (33.3%) |  |
| CD1 | negative | 11 (18.3%) | 1 (33.3%) | 0.476 |
|  | positive | 49 (81.7%) | 2 (66.7%) |  |
| TDT | negative | 8 (13.3%) | 0 (0.0%) | 1.00 |
|  | positive | 52 (86.7%) | 3 (100%) |  |
| HLADR | negative | 48 (80.0%) | 2 (66.7%) | 0.506 |
|  | MHCII | 12 (20.0%) | 1 (33.3%) |  |
| CD117 | negative | 48 (80.0%) | 1 (33.3%) | 0.506 |
|  | positive | 12 (20.0%) | 2 (66.7%) |  |
| MCN | normal | 46 (76.7%) | 0 (0.0%) | **0.001** |
|  | hypodiploidy | 3 (5.0%) | 3 (100%) |  |
|  | hyperdiploidy | 11 (18.3%) | 0 (0.0%) |  |
| Cytogenetics | normal | 20 (33.3%) | 0 (0.0%) | 0.226 |
|  | abnormal | 40 (66.7%) | 3 (100%) |  |
| Complex | negative | 51 (85.0%) | 2 (66.7%) | 0.396 |
|  | positive | 9 (15.0%) | 1 (33.3%) |  |
| MRD15 | <0.01 | 9 (21.4%) | 0 (0.0%) | 0.463 |
|  | >0.01 | 33 (78.6%) | 2 (100%) |  |
| MRD42 | <0.01 | 15 (38.5%) | 0 (0.0%) | 0.541 |
|  | >0.01 | 24 (61.5%) | 3 (100%) |  |
| Relapse | negative | 53 (88.3%) | 2 (66.7%) | 0.339 |
|  | positive | 7 (11.7%) | 1 (33.3%) |  |
| Death | negative | 35 (58.3%) | 1 (33.3%) | 0.572 |
|  | positive | 25 (41.7%) | 2 (66.7%) |  |
| Early death | negative | 49 (81.7%) | 3 (100%) | 0.414 |
|  | positive | 11 (18.3%) | 0 (0.0%) |  |

Data are presented as number (percentage) or median and range. BM: bone marrow, BP: peripheral blood, CD: cluster of differentiation, CR: Complete remission, MCN: modal chromosomal number, MRD: minimal residual disease, TCR: T-cell receptors, TLC: total leukocyte count, WBC: white blood cells count.
