# Supplementary material for: Clinical features, laboratory characteristics, and outcome of ETP and TCRA/D aberrations in pediatric patients with T-acute lymphoblastic leukemia
Source: J Egypt Natl Canc Inst. 2023 Jun 12;35:17. doi: 10.1186/s43046-023-00176-1 (PMC13313959; doi:10.1186/s43046-023-00176-1)
Supplement: Supplementary file 3 — Additional file 3: Supp. 3. Clinical characteristics of relapsed pediatric T-ALL patients. [file 43046_2023_176_MOESM3_ESM.docx]

**Supp. 3:** Clinical characteristics of relapsed pediatric T-ALL patients.

| **No** | **Gender** | **Age**  (years) | **WBC count** (x10^9^/L) | **IPT** | **Cytogenetics** | **TCR abnormality** | **Therapy after relapse** | **DFS** (month) | **OS** (month) |
| --- | --- | --- | --- | --- | --- | --- | --- | --- | --- |
| **1** | Male | 9 | 545.2 | T early | 46,XY[9] | no | FLAG/M | 6 | 71 |
| **2** | Female | 4 | 714.6 | T late | 45, XX, +10,-17,-20 [8] | no | no | 9 | 10 |
| **3** | Male | 2 | 325.1 | T intermediate | 46,XY,der? t(14q11.2;?)[4] | yes | FLAG/M | 19 | 20 |
| **4** | Male | 2.5 | 641.2 | T late | 46,XY[5] | no | no | 6 | 6 |
| **5** | Female | 6 | 406.1 | T early | 46,XX[10] | no | FLAG/M | 14 | 17 |
| **6** | Male | 13 | 100 | ETP | 45,XY,-14[18]/ 46,XY[2] | yes | FLAG/M | 8 | 8 |
| **7** | Female | 6 | 81.53 | T intermediate | 46, XX [20] | no | FLAG/M | 8 | 10 |
| **8** | Male | 6 | 853.9 | T early | 46, XY, del(9p) [20] | no | FLAG/M | 8 | 16 |

DFS: disease free survival, IPT: immunophenotyping, OS: overall survival, TCR: T-cell receptors, WBC: white blood cells count
